# The serum level of irisin, but not asprosin, is abnormal in polycystic ovary syndrome patients

**DOI:** 10.1038/s41598-019-42061-9

**Published:** 2019-04-23

**Authors:** Chia Lin Chang, Shang Yu Huang, Ya Chiung Hsu, Tzu Hsuan Chin, Yung Kuei Soong

**Affiliations:** Department of Obstetrics and Gynecology, Chang Gung Memorial Hospital Linkou Medical Center, Chang Gung University, 5 Fu-Shin Street, Kweishan, Taoyuan, Taiwan

**Keywords:** Endocrine reproductive disorders, Reproductive signs and symptoms

## Abstract

Polycystic ovary syndrome (PCOS) is a disorder characterized by hyperandrogenism, oligo- or anovulation, and/or polycystic ovary. It frequently presents with dyslipidemia and insulin resistance. Recent studies have shown that the white adipose tissue-derived asprosin is elevated in humans with insulin resistance. Because many PCOS patients have a propensity to develop dyslipidemia and/or insulin resistance, asprosin metabolism could be dysregulated in PCOS patients. Accordingly, we investigated serum levels of asprosin, irisin, GIP, androgens, LH, glucose, insulin, and lipids as well as HOMA-IR, QUICKI and ISI _Matsuda_ in a cohort of 444 PCOS patients and 156 controls. Patients were stratified based on metabolic syndrome risk factors (ATPIII [+] and [−] groups), or BMI (overweight and lean groups). The irisin level was significantly correlated with body weight, SBP, DBP, Ferriman–Gallwey score, and levels of TSH, triglycerides, glucose and insulin in the overall population, and was elevated in ATPIII(+) and overweight PCOS patients compared to corresponding controls. By contrast, asprosin levels in PCOS, ATPIII(+), or overweight patients were similar to those of corresponding controls. This finding indicated that the regulation of irisin, but not asprosin, metabolism is abnormal in PCOS patients, and this metabolic characteristic is distinctly different from that of diabetes patients.

## Introduction

Polycystic ovary syndrome (PCOS) is the most common endocrine abnormality of reproductive-age women worldwide^1–3^. Most PCOS patients suffer hyperandrogenism, oligo- or anovulation, and/or hirsutism. They also frequently exhibit dyslipidemia, hyperinsulinism, and elevated LH and anti-Mullerian hormone (AMH) levels^4–7^. Although the molecular etiology of PCOS remains to be clarified, a popular hypothesis considers androgen excess and hyperinsulinemia as primary defects in PCOS patients^8–12^. In addition, dyslipidemia and altered production of sex hormone-binding globulin (SHBG), leptin, and adropin appear to play a role in the pathogenesis of PCOS-associated metabolic dysfunction^13–18^. More recently, we and others have shown that the circulating level of irisin, a newly discovered muscle-derived brown adipose-differentiation factor, is significantly elevated in PCOS patients^15,16^. This finding suggested that, in addition to androgenism and hypersinsulinemia, abnormal regulation of nutritionally responsive hormones such as irisin could contribute to the manifestation of PCOS.

Because many PCOS patients have a tendency to become overweight/obese and develop insulin resistance, we speculated that PCOS could be associated with dysregulation of additional adipogenic and glucogenic hormones. Of interest, recent studies have identified a C-terminal cleavage product of profibrillin (encoded by FBN1 gene), or asprosin, as a white adipose tissue-derived glucogenic adipokine that normally modulates hepatic glucose release^19,20^. It was also shown that asprosin can cross the blood-brain barrier and directly activate orexigenic AgRP+ neurons to inhibit downstream anorexigenic proopiomelanocortin (POMC)-positive neurons, leading to appetite stimulation^21,22^. Importantly, asprosin level is pathologically increased in humans and mice with insulin resistance or type II diabetes (T2D), and in obese humans and mice^21,22^. In addition, blockage of asprosin signaling has a profound glucose- and insulin-lowering effect, suggesting that aberrant regulation of asprosin may contribute to the development of select metabolic diseases, whereas a reduction of asprosin level could be useful for protecting against hyperinsulinism.

Based on these recent findings, we hypothesized that abnormal regulation of asprosin could be associated with the manifestation of PCOS. To investigate this hypothesis, and further examine the hypothesis that irisin is a potential PCOS biomarker, we analyzed serum levels of these hormones in a large cohort of PCOS patients and controls as well as their relationships with select metabolic and endocrine parameters.

## Results

### Levels of fasting irisin, but not asprosin, in PCOS patients were significantly higher than were those in control women

Levels of asprosin, irisin and a variety of metabolic indicators were analyzed in a total of 444 PCOS patients and 156 healthy women who were recruited over a 7-year period (2010–2016). These patients aged from 18 to 34, and the average age of PCOS patients was 2.4 years younger than that of healthy women (PCOS, 25 ± 0.22 yrs vs. controls, 27.42 ± 0.37 yrs; Table 1). Measurements of body weight (BW), body mass index (BMI), body adiposity index (BAI), lean body weight (LBW), systolic blood pressure (SBP), diastolic blood pressure (DBP), waist circumference (WC), hip circumference (HC), WC/HC ratio, and the Ferriman–Gallwey score (F-M score) showed these parameters of PCOS patients were significantly higher than were those of control women. Likewise, levels of free-testosterone, dehydroepiandrosterone (DHEA), androstenedione (ASD), prolactin, LH, testosterone, stimulated glucose, fasting and stimulated insulin, fasting and stimulated C-peptide, Apo-B, HOMA-IR (Homeostatic Model Assessment for Insulin Resistance), AUC(glucose), AUC(insulin), incremental AUC, VLDL-cholesterol, LDL-cholesterol, T-Chol/HDL-Chol ratio, LDL-Chol/HDL-Chol ratio, T-cholesterol, and triglycerides in PCOS patients were significantly higher than were those of control women. On the other hand, levels of FSH and measurements of quantitative insulin-sensitivity check index (QUICKI) and the Matsuda’s sensitivity index (ISI _Matsuda_) of PCOS patients were significantly lower than were those of control patients. The analysis of HOMA-IR, QUICKI, and ISI_matsuda_ indicated that insulin metabolism in PCOS patients was significantly less efficient than was that of control women. Likewise, the irisin level in PCOS patients is significantly elevated as reported earlier (Table 1; *p* < 0.05). However, circulating levels of asprosin and GIP in PCOS patients were not significantly different from those of control women.

**Table 1:** Anthropometric characteristics and metabolic status of PCOS and control patients.

	PCOS (N = 444)	Control (N = 156)	P value
Age	25 ± 0.22	27.42 ± 0.37	**3.9137E-08**
Height (cm)	160.39 ± 0.26	160.74 ± 0.46	0.495203756
BW (kg)	64.84 ± 0.72	58.55 ± 1.01	**4.83036E-06**
BMI	25.23 ± 0.28	22.59 ± 0.36	**5.18494E-07**
BAI	30.41 ± 0.25	28.34 ± 0.32	**1.28034E-05**
LBW	43.91 ± 0.24	42.46 ± 0.39	**0.001757814**
SBP (mm Hg)	116.63 ± 0.73	110.16 ± 1.27	**9.4985E-06**
DBP (mm Hg)	70.41 ± 0.53	66.9 ± 0.77	**0.00054204**
Waist Cir. (cm)	79.74 ± 0.67	73.66 ± 0.90	**1.54911E-06**
Hip Cir. (cm)	98.16 ± 0.47	94.33 ± 0.65	**1.73198E-05**
WC/HC ratio	0.81 ± 0.004	0.78 ± 0.005	**6.51464E-05**
F-M score	8.34 ± 0.22	3.43 ± 0.15	**4.51508E-34**
Free-testo (pg/ml)	1.83 ± 0.04	1.21 ± 0.03	**2.78521E-19**
DHEA (ng/ml)	2410.02 ± 50.22	1994.83 ± 68.61	**1.62934E-05**
ASD (ng/ml)	2.09 ± 0.04	1.48 ± 0.08	**2.12373E-11**
TSH (uIU/mL)	1.71 ± 0.04	1.66 ± 0.07	0.548081324
Prolactin (ng/mL)	12.05 ± 0.25	13.73 ± 0.48	**0.000906978**
LH (mIU/L)	6.98 ± 0.23	5.03 ± 0.27	**4.01534E-06**
FSH (mIU/mL)	5.68 ± 0.09	6.41 ± 0.13	**1.404E-05**
Estradiol (pg/mL)	44.2 ± 2.38	37.52 ± 1.21	0.101916238
Testosterone (ng/mL)	0.56 ± 0.009	0.39 ± 0.01	**1.20751E-21**
Glucose - 0 hr (mg/dL)	83.88 ± 0.66	84.15 ± 1.11	0.834449768
Glucose - 1 hr (mg/dL)	132.38 ± 2.02	118.24 ± 3.48	**0.000410105**
Glucose - 2 hr (mg/dL)	111.89 ± 1.64	103.73 ± 2.84	**0.012209739**
Glucose - 3 hr (mg/dL)	86.86 ± 1.41	81.64 ± 2.18	0.054477145
Insulin - 0 hr (mU/L)	11.22 ± 0.40	6.51 ± 0.27	**3.35032E-11**
Insulin - 1 hr (mU/L)	92.66 ± 3.87	53.76 ± 3.08	**1.49483E-08**
Insulin - 2 hr (mU/L)	81.38 ± 3.63	43.38 ± 2.59	**3.08544E-09**
Insulin - 3 hr (mU/L)	39.21 ± 2.86	17.91 ± 1.39	**1.71766E-05**
C-peptide - 0 hr (ng/ml)	2.04 ± 0.06	1.4 ± 0.06	**1.68009E-09**
C-peptide - 1 hr (ng/ml)	10.07 ± 0.20	7.9 ± 0.25	**3.69994E-09**
Insulin Ab	7.2 ± 0.06	7.06 ± 0.07	0.330236243
Apo-A1	39.2 ± 3.04	24.72 ± 4.37	0.052787348
Apo-B	21.46 ± 1.71	11.15 ± 2.00	**0.011029066**
HOMA-IR	2.43 ± 0.11	1.4 ± 0.08	**2.53662E-07**
QUICKI	0.35 ± 0.002	0.38 ± 0.003	**2.43962E-11**
ISI_matsuda_	7.29 ± 0.27	10.71 ± 0.47	**3.97394E-10**
AUC (glucose)	329.2 ± 4.24	304.61 ± 7.30	**0.003404764**
AUC (insulin)	198.98 ± 8.09	109.29 ± 5.61	**3.58233E-10**
Incremental AUC	0.58 ± 0.02	0.35 ± 0.01	**2.4286E-10**
HDL-cholesterol (mg/dL)	54.17 ± 0.64	56.59 ± 0.98	0.050278502
VLDL-cholesterol (mg/dL)	19.31 ± 0.54	15.48 ± 1.05	**0.000852938**
LDL-cholesterol (mg/dL)	103.44 ± 1.35	96.33 ± 1.82	**0.004990079**
T-Chol/HDL-Chol ratio	3.46 ± 0.05	3.08 ± 0.06	**5.87197E-05**
LDL-Chol/HDL-Chol ratio	2.05 ± 0.04	1.77 ± 0.04	**8.60393E-05**
T-cholesterol (mg/dL)	177.25 ± 1.48	168.28 ± 2.29	**0.001631302**
Triglyceride (mg/dL)	97.77 ± 3.01	77.43 ± 5.61	**0.000864045**
NonHDL-cholesterol (mg/dL)	124.58 ± 1.60	119.37 ± 6.67	0.342726001
Irisin (ng/ml)	789.85 ± 18.41	699.57 ± 27.84	**0.010695875**
GIP (pg/ml)	27.96 ± 1.26	24.65 ± 2.22	0.195450714
Asprosin (ng/ml)	65.72 ± 5.24	50.01 ± 7.47	0.116851096

Significant differences are indicated by bold *p* values.

Analysis of correlations between metabolic parameters and irisin, or asprosin, showed that irisin level was positively correlated with BW, LBW, SBP, LBP, F-M score, TSH, triglycerides, stimulated glucose level, fasting and stimulated insulin levels, stimulated C-peptide level, HOMA-IR, AUC(glucose), AUC(insulin), and incremental AUC, but is negatively associated with QUICKI, ISI_matsuda_ in the overall population (Table S1; *p* < 0.05). On the other hand, asprosin level was only significantly correlated with the level of FSH, free testosterone, and testosterone.

### Irisin, but not asprosin, level was elevated in ATPIII [+] and overweight patients

To determine whether irisin and asprosin levels are related to the metabolic status of patients independent of previously recognized PCOS risk factors such as insulin resistance, obesity and dyslipidemia, we stratified patients based on the presence/absence of ATPIII metabolic syndromes risk factors(s), or BMI^23^. First, we compared metabolic parameters of women with or without ATPIII risk factors in the overall population. Except for levels of DHEA, ASD, prolactin, estradiol, insulin Ab, Apo-A1, and T-cholesterol, all other parameters, including levels of irisin and GIP, of the ATPIII(+) group (N = 295) were significantly different from those of ATPIII(−) group (N = 305) (Table 2). As expected, ATPIII(+) individuals had higher BW, BMI, BAI, LBW, SBP, DBP, WC, HC, WC/HC ratio, F-M score, free testosterone, TSH, testosterone, fasting and stimulated glucose, fasting and stimulated insulin, fasting and stimulated C-peptide, Apo-B, HOMA-IR, AUC(glucose), AUC(insulin), incremental AUC, VLDL-cholesterol, LDL-cholesterol, triglyceride, nonHDL-cholesterol when compared to ATPIII(−) individuals. The ATPIII(+) group had lower levels of LH, FSH, QUICKI, ISI_matsuda_, and HDL-cholesterol when compared with the ATPIII(−) group. The ATPIII(+) individuals also had significantly elevated irisin and GIP levels. On the other hand, the asprosin level was similar in ATPIII(+) and ATPIII(−) subgroups.

**Table 2:** Anthropometric characteristics and metabolic status of ATPIII [−] and ATPIII [+] individuals.

	All ATP(−) (N = 305)	All ATP(+) (N = 295)	P value
Age	24.8 ± 0.28	26.49 ± 0.26	**1.23438E-05**
Height (cm)	161.06 ± 0.31	159.89 ± 0.33	**0.00924475**
BW (kg)	54.29 ± 0.42	72.42 ± 0.88	**3.32303E-62**
BMI	20.93 ± 0.15	28.3 ± 0.32	**9.91472E-73**
BAI	26.97 ± 0.18	32.9 ± 0.29	**9.1227E-55**
LBW	41.56 ± 0.21	45.57 ± 0.31	**5.23173E-25**
SBP (mm Hg)	107.43 ± 0.70	122.6 ± 0.89	**3.26248E-36**
DBP (mm Hg)	64.6 ± 0.43	74.54 ± 0.67	**9.74254E-32**
Waist Cir. (cm)	69.66 ± 0.36	87.04 ± 0.79	**5.24369E-69**
Hip Cir. (cm)	91.79 ± 0.33	102.78 ± 0.56	**3.96353E-53**
WC/HC ratio	0.76 ± 0.003	0.84 ± 0.005	**1.40153E-42**
F-M score	6.32 ± 0.22	7.82 ± 0.30	**5.83024E-05**
Free-testo (pg/ml)	1.48 ± 0.04	1.86 ± 0.04	**8.8883E-10**
DHEA (ng/ml)	2297.33 ± 56.14	2307.42 ± 62.38	0.90563999
ASD (ng/ml)	1.93 ± 0.06	1.94 ± 0.05	0.919129317
TSH (uIU/mL)	1.54 ± 0.05	1.86 ± 0.06	**2.05741E-05**
Prolactin (ng/mL)	12.6 ± 0.32	12.37 ± 0.31	0.603442361
LH (mIU/L)	7.17 ± 0.30	5.75 ± 0.22	**0.000133737**
FSH (mIU/mL)	6.09 ± 0.12	5.63 ± 0.09	**0.001906312**
Estradiol (pg/mL)	42.07 ± 2.27	42.86 ± 2.79	0.826138193
Testosterone (ng/mL)	0.5 ± 0.01	0.54 ± 0.01	**0.013015791**
Glucose - 0 hr (mg/dL)	81.08 ± 0.32	86.92 ± 1.08	**2.16071E-07**
Glucose - 1 hr (mg/dL)	112.32 ± 1.84	145.64 ± 2.71	**8.75251E-23**
Glucose - 2 hr (mg/dL)	97.47 ± 1.16	122.44 ± 2.44	**2.09874E-19**
Glucose - 3 hr (mg/dL)	78.59 ± 1.10	92.63 ± 2.06	**2.35598E-09**
Insulin - 0 hr (mU/L)	6.27 ± 0.20	13.85 ± 0.53	**2.31802E-37**
Insulin - 1 hr (mU/L)	55.14 ± 2.57	110.87 ± 5.11	**2.85988E-21**
Insulin - 2 hr (mU/L)	42.97 ± 1.88	100.86 ± 4.90	**2.43143E-26**
Insulin - 3 hr (mU/L)	18.15 ± 1.10	49.68 ± 4.08	**1.73424E-13**
C-peptide - 0 hr (ng/ml)	1.28 ± 0.03	2.48 ± 0.07	**8.45347E-44**
C-peptide - 1 hr (ng/ml)	8.01 ± 0.19	11.04 ± 0.24	**5.29879E-22**
Insulin Ab	7.18 ± 0.06	7.14 ± 0.07	0.759737347
Apo-A1	33.11 ± 3.65	37.32 ± 3.51	0.527417075
Apo-B	14.6 ± 1.64	22.48 ± 2.18	**0.029020424**
HOMA-IR	1.27 ± 0.04	3.09 ± 0.16	**2.63871E-27**
QUICKI	0.38 ± 0.002	0.34 ± 0.002	**1.26067E-46**
ISI_matsuda_	11.09 ± 0.36	5.16 ± 0.21	**1.0645E-38**
AUC (glucose)	289.05 ± 3.12	357.7 ± 6.16	**4.98812E-22**
AUC (insulin)	110.12 ± 4.40	243.41 ± 10.80	**4.64244E-28**
Incremental AUC	0.37 ± 0.01	0.68 ± 0.03	**6.24633E-22**
HDL-cholesterol (mg/dL)	63.89 ± 0.61	45.41 ± 0.48	**3.60628E-88**
VLDL-cholesterol (mg/dL)	12.27 ± 0.25	24.48 ± 0.82	**1.32384E-38**
LDL-cholesterol (mg/dL)	96.26 ± 1.43	107.12 ± 1.66	**8.7619E-07**
T-Chol/HDL-Chol ratio	2.73 ± 0.03	4.02 ± 0.06	**6.08867E-68**
LDL-Chol/HDL-Chol ratio	1.54 ± 0.02	2.43 ± 0.05	**3.53319E-55**
T-cholesterol (mg/dL)	172.73 ± 1.63	177.17 ± 1.90	0.076759375
Triglyceride (mg/dL)	61.39 ± 1.24	124.63 ± 4.62	**5.36119E-36**
NonHDL-cholesterol (mg/dL)	110.06 ± 1.47	136.23 ± 3.86	**4.95148E-08**
Irisin (ng/ml)	720.58 ± 18.27	814.12 ± 25.98	**0.002512688**
GIP (pg/ml)	24.37 ± 1.32	29.9 ± 1.77	**0.01492013**
Asprosin (ng/ml)	57.51 ± 5.58	66.03 ± 6.70	0.330001375

Significant differences are indicated by bold *p* values.

Similar to analyses based on the ATPIII factor status, analyses based on the BMI status showed that, except for levels of ASD, prolactin, estradiol, and insulin Ab, all other metabolic characteristics in overweight individuals (N = 231) were significantly different from those of lean individuals (N = 368) (Table 3). The level of irisin, but not asprosin, in overweight individuals was significantly higher than that of lean individuals.

**Table 3:** Anthropometric characteristics and metabolic status of lean and overweight individuals.

	All BMI < 25 (N = 368)	All BMI ≥ 25 (N = 231)	P value
Age	25.27 ± 0.25	26.17 ± 0.30	**0.024093219**
Height (cm)	161.08 ± 0.28	159.53 ± 0.36	**0.000807264**
BW (kg)	53.89 ± 0.34	78 ± 0.81	**3.6115E-128**
BMI	20.76 ± 0.11	30.59 ± 0.27	**9.0059E-163**
BAI	26.77 ± 0.14	34.85 ± 0.26	**1.3321E-117**
LBW	41.47 ± 0.19	46.81 ± 0.33	**1.03261E-43**
SBP (mm Hg)	108.7 ± 0.66	124.84 ± 0.98	**9.32916E-39**
DBP (mm Hg)	65.36 ± 0.43	76.07 ± 0.75	**3.8063E-35**
Waist Cir. (cm)	69.69 ± 0.32	91.78 ± 0.74	**2.5385E-126**
Hip Cir. (cm)	91.42 ± 0.27	106.41 ± 0.50	**8.1131E-113**
WC/HC ratio	0.76 ± 0.003	0.86 ± 0.005	**4.97447E-58**
F-M score	6.29 ± 0.21	8.31 ± 0.35	**1.23693E-07**
Free-testo (pg/ml)	1.46 ± 0.03	2.01 ± 0.05	**1.86628E-18**
DHEA (ng/ml)	2237.13 ± 49.56	2416.71 ± 73.91	**0.039657345**
ASD (ng/ml)	1.93 ± 0.05	1.96 ± 0.06	**0.713111154**
TSH (uIU/mL)	1.57 ± 0.04	1.9 ± 0.07	**1.62636E-05**
Prolactin (ng/mL)	12.73 ± 0.29	12.1 ± 0.34	**0.167517085**
LH (mIU/L)	7.17 ± 0.27	5.38 ± 0.22	**2.72939E-06**
FSH (mIU/mL)	6.1 ± 0.10	5.5 ± 0.10	**0.000101055**
Estradiol (pg/mL)	42.4 4± 2.02	42.52 ± 3.36	**0.983061519**
Testosterone (ng/mL)	0.49 ± 0.009	0.57 ± 0.01	**1.40701E-06**
Glucose - 0 hr (mg/dL)	81.63 ± 0.34	87.6 ± 1.34	**2.51111E-07**
Glucose - 1 hr (mg/dL)	116.66 ± 1.82	148.05 ± 3.16	**3.73592E-19**
Glucose - 2 hr (mg/dL)	99.91 ± 1.25	125.44 ± 2.85	**3.51794E-19**
Glucose - 3 hr (mg/dL)	80.55 ± 1.14	93.37 ± 2.41	**1.25584E-07**
Insulin - 0 hr (mU/L)	6.29 ± 0.15	15.89 ± 0.06	**1.63895E-60**
Insulin - 1 hr (mU/L)	56.81 ± 2.28	123.79 ± 6.14	**2.83753E-29**
Insulin - 2 hr (mU/L)	45.49 ± 1.83	112.94 ± 5.84	**2.69263E-34**
Insulin - 3 hr (mU/L)	19.75 ± 0.97	55.88 ± 5.12	**1.54065E-16**
C-peptide - 0 hr (ng/ml)	1.31 ± 0.03	2.77 ± 0.08	**9.52992E-66**
C-peptide - 1 hr (ng/ml)	8.13 ± 0.17	11.71 ± 0.26	**3.5576E-29**
Insulin Ab	7.15 ± 0.06	7.17 ± 0.08	0.865163763
Apo-A1	27.32 ± 3.01	46.51 ± 4.35	**0.004296233**
Apo-B	12.46 ± 1.38	27.4 ± 2.64	**3.83679E-05**
HOMA-IR	1.28 ± 0.03	3.56 ± 0.19	**8.09009E-42**
QUICKI	0.38 ± 0.002	0.33 ± 0.002	**6.36642E-63**
ISI_matsuda_	10.62 ± 0.32	4.3 ± 0.19	**6.52627E-42**
AUC (glucose)	297.17 ± 3.26	363.78 ± 7.30	**1.2912E-19**
AUC (insulin)	115.14 ±± 3.99	272.49 ± 12.94	**1.07916E-37**
Incremental AUC	0.38 ± 0.01	0.75 ± 0.03	**1.30954E-31**
HDL-cholesterol (mg/dL)	60.23 ± 0.65	46.17 ± 0.61	**5.68746E-42**
VLDL-cholesterol (mg/dL)	14.03 ± 0.35	25.11 ± 0.98	**1.93449E-29**
LDL-cholesterol (mg/dL)	96.97 ± 1.39	109.16 ± 1.75	**7.50866E-08**
T-Chol/HDL-Chol ratio	2.92 ± 0.04	4.07 ± 0.07	**2.55004E-48**
LDL-Chol/HDL-Chol ratio	1.68 ± 0.03	2.47 ± 0.05	**8.64712E-39**
T-cholesterol (mg/dL)	171.12 ± 1.62	181.2 ± 1.91	**8.03673E-05**
Triglyceride (mg/dL)	69.62 ± 1.74	129.07 ± 5.63	**1.05343E-29**
NonHDL-cholesterol (mg/dL)	115.58 ± 3.15	134.99 ± 2.09	**8.91048E-05**
Irisin (ng/ml)	735.09 ± 18.12	815.09 ± 27.77	**0.012067818**
GIP (pg/ml)	24.22 ± 1.23	31.57 ± 2.04	**0.001565489**
Asprosin (ng/ml)	58.49 ± 5.26	65.63 ± 7.48	**0.425414846**

Significant differences are indicated by bold *p* values.

Because serum irisin level was affected by the metabolic status of patients, we conducted partial correlation analysis in order to better explain the relationships between irisin level and different metabolic/endocrine parameters. As shown in Table S1, the irisin level was significantly correlated with 19 variables in the overall population. Partial correlation analysis showed that the correlation between irisin level and each of these variables becomes nonsignificant when the other 18 variables were used as controlling variables (Table S2). Because BW is one of the most possible confounding factors, we also performed partial correlation analysis with the BW as the only controlling variable. This analysis showed the correlations between irisin level and TSH level, or ISI _Matsuda_, remain significant (Table S2).

### The irisin, but not asprosin, level was elevated in ATPIII [+] and overweight PCOS patients when compared to ATPIII(−) and lean PCOS patients, respectively

**Figure 1:**
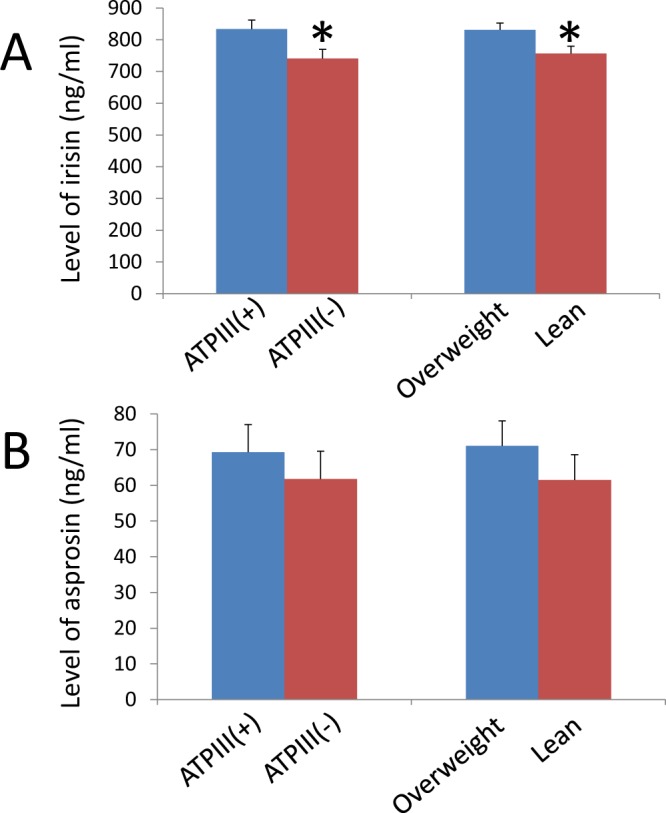
Fasting irisin, but not asprosin, levels are significantly elevated in ATPIII(+) and overweight PCOS patients. Levels of irisin (**A**), but not asprosin (**B**), were significantly elevated in ATPIII(+) and overweight PCOS patients when compared to those of ATPIII(−) and lean PCOS patients, respectively. *Significantly different between the subgroups (*p* < 0.05).

Comparison of metabolic parameters between ATPIII(+) PCOS and ATPIII(−) PCOS patients showed that ATPIII(+) PCOS patients (N = 235) were characterized by higher levels of BMI, BAI, LBW, SBP, DBP, WC, HC, WC/HC ratio, F-M score, free testosterone, TSH, fasting and stimulated glucose, fasting and stimulated insulin, fasting and stimulated C-peptide, HOMA-IR, AUC(glucose), AUC(insulin), LDL cholesterol, triglyceride, irisin, and GIP when compared to ATPIII(−) PCOS patients (N = 209)(Table 4; Fig. 1). On the other hand, levels of asprosin, DHEA, ASD, prolactin, estradiol, and testosterone were not significantly different between the two groups. Comparisons of ATPIII [−] PCOS patients (N = 209) and ATPIII [−] controls (N = 96), who shared similar anthropometric characteristics, showed major differences between these two subcohorts were levels of LH, testosterone, fasting and stimulated insulin, fasting and stimulated C-peptide, and AUC(insulin) as well as the F-M score (Table S3). The difference in irisin level between ATPIII(−) PCOS and ATPIII(−) control patients was on the border of significance (*p* = 0.10).

**Table 4:** Anthropometric characteristics and metabolic status of ATPIII(−) and ATPIII(+) PCOS patients.

	PCOS with ATP(−) (N = 209)	PCOS with ATP(+) (N = 235)	P value
Age	24.02 ± 0.33	25.88 ± 0.29	**2.35233E-05**
Height (cm)	161.36 ± 0.35	159.53 ± 0,37	**0.000380497**
BW (kg)	54.51 ± 0.49	74.03 ± 0.96	**4.81355E-52**
BMI	20.95 ± 0.18	29.04 ± 0.35	**4.01821E-63**
BAI	26.89 ± 0.22	33.56 ± 0.32	**4.19262E-49**
LBW	41.76 ± 0.23	45.81 ± 0.35	**2.84409E-19**
SBP (mm Hg)	108.08 ± 0.70	124.15 ± 1.00	**5.66316E-32**
DBP (mm Hg)	64.63 ± 0.49	75.5 ± 0.77	**3.76296E-27**
Waist Cir. (cm)	69.85 ± 0.44	88.61 ± 0.86	**1.40353E-57**
Hip Cir. (cm)	91.87 ± 0.38	103.8 ± 0.63	**7.75126E-45**
WC/HC ratio	0.76 ± 0.005	0.85 ± 0.005	**2.41987E-34**
F-M score	7.57 ± 0.27	9.02 ± 0.33	**0.000849631**
Free-testo (pg/ml)	1.62 ± 0.05	2.02 ± 0.05	**8.86558E-08**
DHEA (ng/ml)	2424.27 ± 70.42	2397.46 ± 71.40	0.79328654
ASD (ng/ml)	2.09 ± 0.06	2. 1± 0.06	0.923624747
TSH (uIU/mL)	1.49 ± 0.06	1.9 ± 0.07	**4.62543E-06**
Prolactin (ng/mL)	11.94 ± 0.36	12.16 ± 0.04	0.647335166
LH (mIU/L)	8 ± 0.38	6.09 ± 0.25	**3.03239E-05**
FSH (mIU/mL)	5.91 ± 0.15	5.47 ± 0.10	**0.013038594**
Estradiol (pg/mL)	43.64 ± 3.25	44.7 ± 3.45	0.825805461
Testosterone (ng/mL)	0.54 ± 0.01	0.5 8± 0.01	0.07922658
Glucose - 0 hr (mg/dL)	80.86 ± 0.39	86.56 ± 1.18	**1.46781E-05**
Glucose - 1 hr (mg/dL)	114.08 ± 2.26	148.66 ± 2.85	**4.04834E-19**
Glucose - 2 hr (mg/dL)	98.44 ± 1.45	123.8 ± 2.59	**1.80309E-15**
Glucose - 3 hr (mg/dL)	79.79 ± 1.38	93.15 ± 2.28	**1.688E-06**
Insulin - 0 hr (mU/L)	6.71 ± 0.26	15.24 ± 0.62	**7.43154E-30**
Insulin - 1 hr (mU/L)	59.67 ± 3.45	121.99 ± 6.02	**6.67615E-17**
Insulin - 2 hr (mU/L)	46.95 ± 2.56	111.84 ± 5.78	**9.34794E-21**
Insulin - 3 hr (mU/L)	20.32 ± 1.49	56 ± 5.00	**2.25712E-10**
C-peptide - 0 hr (ng/ml)	1.33 ± 0.04	2.6 6 ± 0.08	**1.37062E-35**
C-peptide - 1 hr (ng/ml)	8.32 ± 0.25	11.62 ± 0.26	**2.2959E-18**
Insulin Ab	7.24 ± 0.08	7.16 ± 0.08	0.599841354
Apo-A1	38.11 ± 4.70	40.09 ± 3.99	0.807151472
Apo-B	17.13 ± 2.14	24.95 ± 2.57	0.086013092
HOMA-IR	1.35 ± 0.05	3.39 ± 0.19	**5.0268E-21**
QUICKI	0.38 ± 0.002	0.33 ± 0.002	**1.01184E-40**
ISI_matsuda_	10.48 ± 0.45	4.45 ± 0.19	**1.63193E-32**
AUC (glucose)	292.18 ± 3.91	362.12 ± 6.51	**1.12458E-17**
AUC (insulin)	119.87 ± 5.96	269.33 ± 12.70	**3.15595E-22**
Incremental AUC	0.4 ± 0.02	0.74 ± 0.03	**1.8834E-17**
HDL-cholesterol (mg/dL)	64.45 ± 0.74	45.03 ± 0.54	**8.33138E-71**
VLDL-cholesterol (mg/dL)	12.48 ± 0.31	25.42 ± 0.80	**1.00535E-36**
LDL-cholesterol (mg/dL)	96.34 ± 1.81	109.78 ± 1.89	**5.33286E-07**
T-Chol/HDL-Chol ratio	2.72 ± 0.04	4.12± 0.07	**9.28802E-56**
LDL-Chol/HDL-Chol ratio	1.53 ± 0.03	2.52 ± 0.05	**2.35092E-44**
T-cholesterol (mg/dL)	173.68 ± 2.01	180.42 ± 2.13	**0.022639457**
Triglyceride (mg/dL)	62.11 ± 1.53	129.49 ± 4.64	**1.5224E-33**
NonHDL-cholesterol (mg/dL)	111.44 ± 1.86	136.09 ± 2.27	**5.94085E-12**
Irisin (ng/ml)	740.9 ± 21.60	833.39 ± 28.74	**0.01198301**
GIP (pg/ml)	24.82 ± 1.25	30.76 ± 2.10	**0.023676321**
Asprosin (ng/ml)	61.73 ± 7.01	69.3 ± 7.71	0.472918322

Significant differences are indicated by bold *p* values.

Likewise, comparisons between overweight PCOS patients (N = 198) and lean PCOS patients (N = 246) showed that, except for levels of DHEA, ASD, prolactin, estradiol, insulin Ab, Apo-A1, and asprosin, levels of other metabolic parameters in overweight PCOS patients were significantly different from those of lean PCOS patients (Table 5; Fig. 1). On the other hand, while major anthropometric measurements and asprosin level are similar in lean PCOS and lean control patients (Table S4), the F-M score and levels of androgens, DHEA, ASD, LH, fasting and stimulated insulin, fasting and stimulated C-peptide, HOMA-IR, AUC(insulin), incremental AUC, VLDL-cholesterol, T-cholesterol, and triglyceride in lean PCOS patients were significantly higher than were those of lean control patients. Similar to the comparison between ATPIII(−) PCOS and ATPIII(−) control patients (Table S3), the difference in irisin level between lean PCOS and lean control patients was on the border of significance (*p* = 0.089) (Table S4). Thus, the PCOS-associated elevation of irisin level was more obvious in ATPIII(+) and overweight PCOS patients when compared to ATPIII(−) and lean PCOS patients, respectively.

**Table 5:** Anthropometric characteristics and metabolic status of lean and overweight PCOS patients.

	PCOS with BMI < 25 (N = 246)	PCOS with BMI ≥ 25 (N = 198)	P value
Age	24.43 ± 0.33	25.72 ± 0.29	**0.003561875**
Height (cm)	161.13 ± 0.36	159.48 ± 0.37	**0.001438865**
BW (kg)	53.97 ± 0.43	78.35 ± 0.79	**2.43754E-97**
BMI	20.78 ± 0.14	30.76 ± 0.27	**6.3506E-121**
BAI	26.76 ± 0.19	34.98 ± 0.27	**3.98975E-86**
LBW	41.51 ± 0.24	46.88 ± 0.32	**1.02861E-34**
SBP (mm Hg)	109.04 ± 0.78	125.99 ± 0.95	**7.85786E-36**
DBP (mm Hg)	65.44 ± 0.55	76.56 ± 0.75	**2.49198E-28**
Waist Cir. (cm)	69.83 ± 0.43	92.18 ± 0.73	**2.47944E-94**
Hip Cir. (cm)	91.44 ± 0.34	106.6 ± 0.52	**1.17721E-84**
WC/HC ratio	0.76 ± 0.004	0.86 ± 0.005	**5.54407E-43**
F-M score	7.67 ± 0.27	9.17 ± 0.34	**0.000569541**
Free-testo (pg/ml)	1. 6 ± 0.05	2.12 ± 0.05	**4.04169E-12**
DHEA (ng/ml)	2363.33 ± 67.56	2469.25 ± 75.22	0.30272413
ASD (ng/ml)	2.11 ± 0.06	2.08 ± 0.06	0.748124981
TSH (uIU/mL)	1.53 ± 0.06	1.93 ± 0.07	**7.58835E-06**
Prolactin (ng/mL)	12.29 ± 0.37	11.76 ± 0.33	0.277771277
LH (mIU/L)	8.04 ± 0.38	5.68 ± 0.22	**2.19205E-07**
FSH (mIU/mL)	5.87 ± 0.15	5.44 ± 0.09	**0.015616852**
Estradiol (pg/mL)	44.09 ± 3.19	44.34 ± 3.58	0.95835939
Testosterone (ng/mL)	0.53 ± 0.012	0.6 ± 0.01	**0.000590776**
Glucose - 0hr (mg/dL)	81.37 ± 0.46	86.99 ± 1.25	**2.07992E-05**
Glucose - 1hr (mg/dL)	118.81 ± 2.41	149.24 ± 2.94	**9.51529E-15**
Glucose - 2hr (mg/dL)	100.73 ± 1.70	125.71 ± 2.61	**6.57559E-15**
Glucose - 3hr (mg/dL)	81.67 ± 1.62	93.32 ± 2.27	**3.43114E-05**
Insulin - 0hr (mU/L)	6.7 ± 0.22	16.85 ± 0.62	**3.61969E-44**
Insulin - 1hr (mU/L)	61.46 ± 3.33	131.42 ± 6.29	**3.8538E-21**
Insulin - 2hr (mU/L)	49.18 ± 2.63	121.22 ± 5.99	**1.32717E-25**
Insulin - 3hr (mU/L)	21.93 ± 1.40	60.69 ± 5.39	**5.60764E-12**
C-peptide - 0hr (ng/ml)	1.35 ± 0.04	2.88 ± 0.08	**6.2134E-50**
C-peptide - 1hr (ng/ml)	8.49 ± 0.24	12.03 ± 0.26	**5.3713E-21**
Insulin Ab	7.18 ± 0.08	7.22 ± 0.09	0.758951171
Apo-A1	31.82 ± 4.30	47.13 ± 4.27	0.05764904
Apo-B	14.94 ± 2.03	28.46 ± 2.67	**0.00267365**
HOMA-IR	1.36 ± 0.05	3.76 ± 0.20	**1.558E-29**
QUICKI	0.37 ± 0.002	0.33 ± 0.002	**9.61963E-51**
ISI_matsuda_	9.97 ± 0.43	3.95 ± 0.17	**5.4465E-32**
AUC(glucose)	300.48 ± 4.45	364.87 ± 6.68	**6.50479E-15**
AUC(insulin)	124.7 ± 5.77	291.25 ± 13.26	**1.08869E-27**
Incremental AUC	0.41 ± 0.02	0.8 ± 0.03	**6.80801E-23**
HDL-cholesterol (mg/dL)	61.02 ± 0.88	45.67 ± 0.59	**1.86139E-38**
VLDL-cholesterol (mg/dL)	14.64 ± 0.52	25.1 ± 0.83	**8.50547E-23**
LDL-cholesterol (mg/dL)	97.94 ± 1.95	110.31 ± 1.79	**4.59155E-06**
T-Chol/HDL-Chol ratio	2.93 ± 0.05	4.12 ± 0.07	**4.97903E-37**
LDL-Chol/HDL-Chol ratio	1.68 ± 0.04	2.52 ± 0.05	**3.70259E-30**
T-cholesterol (mg/dL)	173.43 ± 2.19	181.99 ± 1.94	**0.003841787**
Triglyceride (mg/dL)	72.41 ± 2.52	129.29 ± 4.89	**4.69157E-23**
NonHDL-cholesterol (mg/dL)	114.96 ± 2.18	136.4 ± 2.12	**3.42505E-09**
Irisin (ng/ml)	756.81 ± 23.37	830.91± 28.68	**0.045291804**
GIP (pg/ml)	25.1 ± 1.74	31.34 ± 1.82	**0.017787063**
Asprosin (ng/ml)	61.5 ± 7.09	71.04 ±7.81	0.368453089

Significant differences are indicated by bold *p* values.

## Discussion

The present study showed that serum irisin level is associated with fasting and stimulated glucose and insulin levels, and PCOS patients have an overtly elevated irisin level. In addition, irisin levels in ATPIII(+) and overweight patients are significantly higher than are those in ATPIII(−) or lean patients, respectively. This alteration is concurrent with the central presentation of PCOS (i.e., hyperandrogenism and elevated LH levels). By contrast, the level of asprosin in PCOS patients does not differ from that of controls regardless of the metabolic status of patients. Also, the serum level of asprosin is not associated with comorbidities such as insulin resistance, dyslipidemia or hyperandrogenism in PCOS patients. These data affirmed that PCOS patients are characterized by an elevated irisin, but not asprosin level.

Hepatic glucose release into the circulation is modulated by a spectrum of hormones, and is vital for normal brain function and individual survival during periods of hunger^19,20^. Recently, two newly identified hormones, irisin and asprosin, were shown to regulate glucose and lipid metabolism as well as the development of metabolism disorders. Asprosin was identified as a fasting-induced hormone that is generated from the C-terminus of profibrillin and promotes hepatic glucose production^19,20^. A high level of asprosin is associated with insulin resistance in T2D patients and mice, and the neutralization of asprosin reduces appetite and body weight in obese mice. Asprosin is capable of crossing the blood-brain barrier to act on orexigenic AgRP+ neurons, leading to inhibition of anorexigenic POMC-positive neurons and appetite stimulation^21^. Based on these findings, it has been suggested that asprosin represents a new target for the treatment of T2D. Because serum asprosin level in PCOS patients does not differ from that of healthy individuals, the observed dysregulation of asprosin in T2D patients could be disease-specific even though PCOS patients share similar glucose/insulin and lipid profiles with T2D patients.

The brown adipose-derived irisin was shown to exert beneficial effect of exercise in humans and animals. Recent study indicated that irisin may exert its action in bone and adipose tissues by binding to the αV class of integrins^24^. Although contradictory data on the role of irisin in a number of conditions have been reported^25–27^, a few consistent trends have emerged. First, there is a positive relationship between irisin and a healthy metabolic status, perhaps via the regulation of ERK and AMPK signaling^28–41^. Second, patients with T2D or gestational diabetes mellitus (GDM), but not T1D, are characterized by a low irisin level^25–27,34,42–50^. In addition, maternal irisin levels in patients with early- or late-onset preeclampsia are lower than that of healthy pregnant women^51^. Third, we and others have reported that irisin level is significantly elevated in PCOS patients^52–58^. Consistently, the present study showed that irisin levels are significantly elevated in a large cohort of PCOS patients, and this difference is more obvious in ATPIII(+) and overweight PCOS patients. However, the partial correlation analysis indicated that the significant correlations between irisin level and various metabolic and endocrine variables are interrelated and can be partially explained by the difference in BW. Likewise, a recent meta-analysis had suggested that while circulating irisin level in PCOS patients was higher than that in overall healthy controls, this association could be partly attributed to the BMI of PCOS patients^59^. Nonetheless, the significant difference in irisin levels between ATPIII(+) and overweight PCOS patients and their corresponding controls suggested that the irisin level indeed has a tendency to increase in PCOS patients. On the other hand, it has been shown that the commercially available assay kits for irisin have high coefficients of variation^60^. Therefore, further studies with different assay techniques, and a larger and better defined patient population are needed to verify the present findings.

It was estimated that >42% of PCOS patients in the United States are overweight or obese, and have a high risk of developing T2D, atherosclerosis, and cardiovascular events^7,61–63^. Unlike T2D, which is associated with low irisin and high asprosin levels, PCOS patients are characterized by a high irisin level and a normal asprosin level. These findings suggested the mechanisms underlying insulin resistance and dyslipidemia in T2D and PCOS patients could be rather distinct. It has been speculated that the elevated asprosin level in T2D patients could contribute to the altered metabolic status in patients partly through asprosin’s effects on appetite^19–22^. Because we did not observe significant relationships between asprosin level and major metabolic parameters in PCOS patients, the asprosin-mediated orexigenic response would unlikely represent part of the mechanism underlying metabolic dysregulation in PCOS patients.

Although the origin of elevated irisin level in PCOS patients remains to be investigated, we speculate this aberration could be partly attributed to the altered metabolic status of patients (e.g., elevated LH, androgen, BW, and insulin resistance). Alternatively, the elevated irisin level in PCOS patients may represent a protective mechanism to counteract excess energy inflow^33^, or represent an “irisin resistance” state in which a high irisin level fails to induce a desired physiologic response in PCOS patients^64,65^.

Taken together, our study indicated that the regulation of brown adipose-differentiation factor irisin, but not the white adipose-derived asprosin, represents a potential biomarker for PCOS. Because T2D patients is accompanied by an increased asprosin level, the lack of an association between asprosin and various metabolic parameters in PCOS patients suggests the mechanisms underlying glucose/insulin dysregulation in PCOS and T2D are distinct, and the interplay of irisin, asprosin, and other endocrine hormones could be key to the manifestation of distinct metabolic profiles in PCOS and T2D patients.

## Conclusions

Our finding showed that PCOS patients are characterized by an elevated serum irisin level; whereas the serum asprosin level is not associated with the metabolic manifestation in PCOS patients.

## Materials and Methods

### Subjects and anthropometric measurements

All studies were conducted with approvals from the Human Research Ethics Committee of Chang Gung Memorial Hospital. Informed consent forms were obtained from each participant before beginning the research. All research and methods were performed in accordance with relevant guidelines/regulations as approved by the Human Research Ethics Committee of Chang Gung Memorial Hospital. Control cases were healthy volunteers with regular menstruation. Women with PCOS were diagnosed based on the revised Rotterdam criteria, which require two of the following three manifestations: (1) oligo- and/or anovulation, (2) clinical and/or biochemical hyperandrogenism, and (3) polycystic ovaries^66^. Current smokers and those who consumed alcohol or took medicines affecting glucose/lipid/androgen metabolism during the 6 months before enrollment were excluded. These exclusion criteria were chosen to avoid the interference of oral contraceptives and insulin-sensitizing drugs^7^. Additional exclusion criteria included hypothyroidism, hyperthyroidism, congenital adrenal hyperplasia, Cushing’s syndrome, hyperprolactinemia and androgen-secreting tumors.

A total of 444 PCOS patients and 156 healthy women aged 18 to 34 were enrolled for the present study. Anthropometric measurements, including age, body weight (BW), body mass index (BMI), body adiposity index (BAI), lean body weight (LBW), systolic blood pressure (SBP), diastolic blood pressure (DBP), waist circumference (WC), and hip circumference (HC), were measured following standard procedures^67,68^. Hirsutism was measured with a modified version of the Ferriman–Gallwey evaluation system.

### Oral glucose tolerance test and assays of metabolic biomarkers

To determine the enteroinsular response, a 3-hr oral glucose tolerance test (OGTT) was carried out using 75 g of glucose (Glucola^TM^) after an 8–12 hr overnight fast. All subjects were advised to follow their normal diet for at least 3 days prior to the test. The experiment was conducted on Day 2–4 of the menstrual cycle, and blood samples were drawn from the antecubital vein at 0, 1, 2 and 3 hr after glucose intake, and stored at −20C before the analysis.

Glucose and insulin levels were measured at 0, 1, 2 and 3 hr following glucose intake. The HOMA-IR (Homeostatic Model Assessment for Insulin Resistance) and quantitative insulin-sensitivity check index (QUICKI) were used to assess insulin resistance and insulin sensitivity^69^. Peripheral insulin resistance was assessed by the Matsuda’s sensitivity index (ISI _Matsuda_). Areas under the curve for plasma glucose response (AUC(glucose)) and areas under the curve for plasma insulin response (AUC(insulin)) were calculated with the trapezoid rule.

Asprosin, irisin, and GIP were determined by specific enzyme-linked immunoassays (EIAab, Phoenix Pharmaceuticals, and Millipore Corporation). Insulin and testosterone were determined by specific Architect assays, and free-testosterone was determined by a specific radioimmunoassay from Beckman Coulter. Serum dehydroepiandrosterone (DHEA), androstenedione (ASD), estradiol, triglycerides, total cholesterol (T-cholesterol), HDL-cholesterol, VLDL-cholesterol, LDL-cholesterol, glucose, C-peptide, Apo-A1, Apo-B, insulin Ab, FSH, and LH were determined by standard laboratory procedures.

### Subgrouping of subjects

To identify potential biomarkers that are tied to select subgroups of patients, we subcategorized PCOS patients and healthy women according to the BMI or the presence/absence of metabolic syndromes risk factors(s) as defined by the National Cholesterol Education Program’s Adult Treatment Panel III (ATPIII) report^23^. Using the BMI, we subdivided patients into overweight (BMI ≥25) and lean (BMI <25) subcohorts. As for ATPIII factors, we subdivided patients into ATPIII positive (ATPIII [+]) and ATPIII negative (ATPIII [−]) subcohorts. In the ATPIII [+] subcohort, all patients have at least one of the ATPIII risk factors known to contribute to the development of metabolic syndromes.

### Statistical analysis

One-way ANOVA, Student’s *t*-test, and correlation coefficient analyses were conducted using the SPSS statistical software (SPSS Inc., USA). Two-tailed *p*-values < 0.05 were considered statistically significant. Data in Tables S1 and S2 were obtained using the correlation analysis. The pair-wise comparisons between subgroups of patients in other Tables were obtained based on the two-tailed Student’s *t*-test.

## Supplementary Information

### Supplementary information

Supplementary information

### Supplementary information
