# Supplementary material for: The serum level of irisin, but not asprosin, is abnormal in polycystic ovary syndrome patients
Source: Sci Rep. 2019 Apr 23;9:6447. doi: 10.1038/s41598-019-42061-9 (PMC6558936; doi:10.1038/s41598-019-42061-9)
Supplement: Supplementary file 1 — Supplementary information [file 41598_2019_42061_MOESM1_ESM.docx]

**Table S1. Correlations of metabolic parameters with irisin or asprosin in the overall population.** Significant correlations are indicated by bold *p* values.

**Table S2. Partial correlation analysis of the relationships between irisin level and metabolic/endocrine parameters in the overall population.** Significant differences are indicated by bold *p* values.

**Table S3. Anthropometric characteristics and metabolic status of ATPIII(-) PCOS and ATPIII(-) control patients.** Significant differences are indicated by bold *p* values.


**Table S4. Anthropometric characteristics and metabolic status of lean PCOS and lean control patients.** Significant differences are indicated by b
